# *JCEM Case Reports*: A Remarkable First Year

**DOI:** 10.1210/jcemcr/luae007

**Published:** 2024-01-27

**Authors:** William F Young

**Affiliations:** Division of Endocrinology, Diabetes, Metabolism, and Nutrition, Mayo Clinic, Rochester, MN 55905, USA

In January 2023, the Endocrine Society launched *JCEM Case Reports,* a new, open-access, online-only journal. Our mission is to provide a forum for internal medicine residents, endocrine trainees, junior faculty, and clinicians in practice to publish their challenging clinical cases—either as a case report or an image in endocrinology. With the journal launch, we emphasized that a good case report must be factual, concise, well-organized, presented clearly, based on logical causality, and easily readable. To facilitate optimal and uniform submissions, a manuscript template and instructional video (https://academic.oup.com/jcemcr) was developed to guide authors on providing a complete description of their case, pertinent laboratory results and images, focused discussion, and key learning points.

Our goal is to publish educational clinical cases that are well-described, have clear learning points, and are of special significance to early-career endocrinologists and members of endocrine care teams. We are particularly interested in strategies to diagnose and treat endocrine conditions in regions with limited clinical resources; these cases may have important implications for a wider audience. We aim for *JCEM Case Reports* to be the preferred forum to share complex cases and disseminate valuable clinical pearls to busy clinicians. Far too often, the clinical pearls that we learn from managing difficult clinical situations are never shared. *JCEM Case Reports* is the platform for clinicians to pass on these important clinical insights.

With a continuous publication model, we published 6 bimonthly issues in 2023. There were 188 articles published online: 176 case reports, 10 images in endocrinology, 1 editorial, and 1 letter to the editor. The domain distribution of the published case reports in 2023 was diabetes/hypoglycemia/lipids/obesity (18%), adrenal (17%), pituitary/hypothalamus (16%), pediatrics (14%), bone/calcium (13%), thyroid (11%), endocrine syndromes (7%), and reproduction (4%). The overall acceptance rate for manuscripts submitted for publication in 2023 was 57%.

From the beginning, *JCEM Case Reports* has had an international footprint. Our associate editors are from 3 continents and 6 countries. All peer review is performed by our editorial board comprised of 180 members from 34 different countries. In 2023, we received manuscript submissions from 46 different countries, including the United States (46%), Japan (8%), India (7%), and Australia (4%). Recognizing that many article submissions are from clinicians in training, our editorial board and associate editors are charged with evaluating the methodologic quality of case reports and working with authors to optimize their submissions with respect to case selection, ascertainment, causality, and reporting. All published content is free for reading worldwide.

At the 2023 Annual Endocrine Society Meeting, we celebrated our first 6 months of publication by hosting a symposium entitled “Clinical Pearls from *JCEM Case Reports*.” Four authors presented their cases and addressed questions from the audience. Following each presentation, an experienced content expert shared their clinical pearls. A recording of this session is available on the journal website (https://academic.oup.com/jcemcr). To mark our one-year anniversary, we hosted a pituitary-focused online seminar, “Clinical Pearls from *JCEM Case Reports:* Pituitary Edition” (available for viewing at: https://academic.oup.com/jcemcr). *JCEM Case Reports* will host another “Clinical Pearls from *JCEM Case Reports*” symposium at the 2024 Annual Endocrine Society Meeting in Boston, in June 2024, and we hope to see you there!

By all metrics, we have had a successful first year of publication. In 2024, we will maintain our continuous publication model and expand to 12 monthly issues. We hope that you have enjoyed reading the journal, and we look forward to receiving your case report manuscripts!

